# Severe peripartum cardiomyopathy complicated by multiple left ventricular thrombi and systemic embolic events: a case report

**DOI:** 10.1093/ehjcr/ytag685

**Published:** 2026-09-11

**Authors:** Fadoua Achour, Soukaina Cherkaoui, Moustapha Atteyeh Sougal, Jamila Zarzour, Mohamed Cherti

**Affiliations:** Department of Cardiology, Ibn Sina University Hospital, Mohammed V University, Rabat, Morocco; Department of Cardiology, Ibn Sina University Hospital, Mohammed V University, Rabat, Morocco; Department of Cardiology, Ibn Sina University Hospital, Mohammed V University, Rabat, Morocco; Department of Cardiology, Ibn Sina University Hospital, Mohammed V University, Rabat, Morocco; Department of Cardiology, Ibn Sina University Hospital, Mohammed V University, Rabat, Morocco

**Keywords:** Peripartum cardiomyopathy, Left ventricular thrombus, Systemic embolization, Heart failure, Postpartum, Case report

## Abstract

**Background:**

Peripartum cardiomyopathy (PPCM) is a potentially life-threatening cause of heart failure occurring towards the end of pregnancy or in the months following delivery. Severe PPCM may be complicated by intracardiac thrombi and systemic embolization, favoured by left ventricular (LV) systolic dysfunction, ventricular dilatation, and the hypercoagulable state of the puerperium.

**Case summary:**

A 36-year-old woman presented 1-month postpartum with acute decompensated heart failure. Transthoracic echocardiography showed severe LV systolic dysfunction, with a LV ejection fraction (LVEF) of 21%. Computed tomography demonstrated two large LV thrombi and systemic embolic complications involving the spleen, kidneys, and liver, as well as splenic vein thrombosis. She was treated with guideline-directed heart failure therapy and therapeutic anticoagulation. Serial follow-up showed progressive LV recovery and thrombus regression, with complete normalization of LVEF and full thrombus resolution by 8 months.

**Discussion:**

Severe PPCM should be considered a potentially thromboembolic disease, particularly in the presence of marked LV dysfunction or atypical extracardiac symptoms. This case emphasizes the importance of early recognition, systematic assessment for intracardiac and extracardiac thromboembolic complications, individualized anticoagulation, and close imaging follow-up.

Learning points

- Severe peripartum cardiomyopathy (PPCM) may present with extensive thromboembolic complications, especially when left ventricular dysfunction is marked.
- Abdominal symptoms in severe PPCM should prompt consideration of visceral embolization, not only congestion.
- Anticoagulation should be therapeutic and guided by serial imaging until thrombus resolution and ventricular recovery.

## Introduction

Peripartum cardiomyopathy (PPCM) is an uncommon but potentially life-threatening form of heart failure occurring towards the end of pregnancy or in the months following delivery. It is defined by new-onset left ventricular (LV) systolic dysfunction in women without previously known structural heart disease or another identifiable cause of heart failure.^1,2^ Although myocardial recovery may occur, the initial presentation is highly variable, ranging from mild symptoms to severe heart failure, cardiogenic shock, arrhythmias, thromboembolic complications, or persistent LV dysfunction.^1,2^

Timely diagnosis remains challenging because symptoms, such as dyspnoea, fatigue, orthopnoea, and peripheral oedema, may overlap with physiological changes of late pregnancy and the early postpartum period. As a result, PPCM may be recognized only at the stage of advanced heart failure or when complications have already occurred.^1,2^

Beyond ventricular dysfunction, PPCM is associated with a clinically relevant thromboembolic risk. Severe LV systolic impairment, ventricular dilatation, intracavitary stasis, endothelial dysfunction, and the hypercoagulable state of the puerperium may act together to promote intracardiac thrombus formation and embolic events.^1,3^ While intracardiac thrombi and systemic embolization are recognized complications, multivisceral embolization involving several abdominal organs remains uncommon and may be clinically misleading, particularly when abdominal symptoms coexist with congestion, ascites, or hepatic dysfunction.^4–7^

We describe a severe form of PPCM presenting 1 month postpartum with multiple mobile LV thrombi, systemic embolization involving the kidneys, spleen, and liver, and associated splenic vein thrombosis, followed by complete LV recovery and thrombus resolution under heart failure therapy and therapeutic anticoagulation.

## Summary figure

**Figure:**
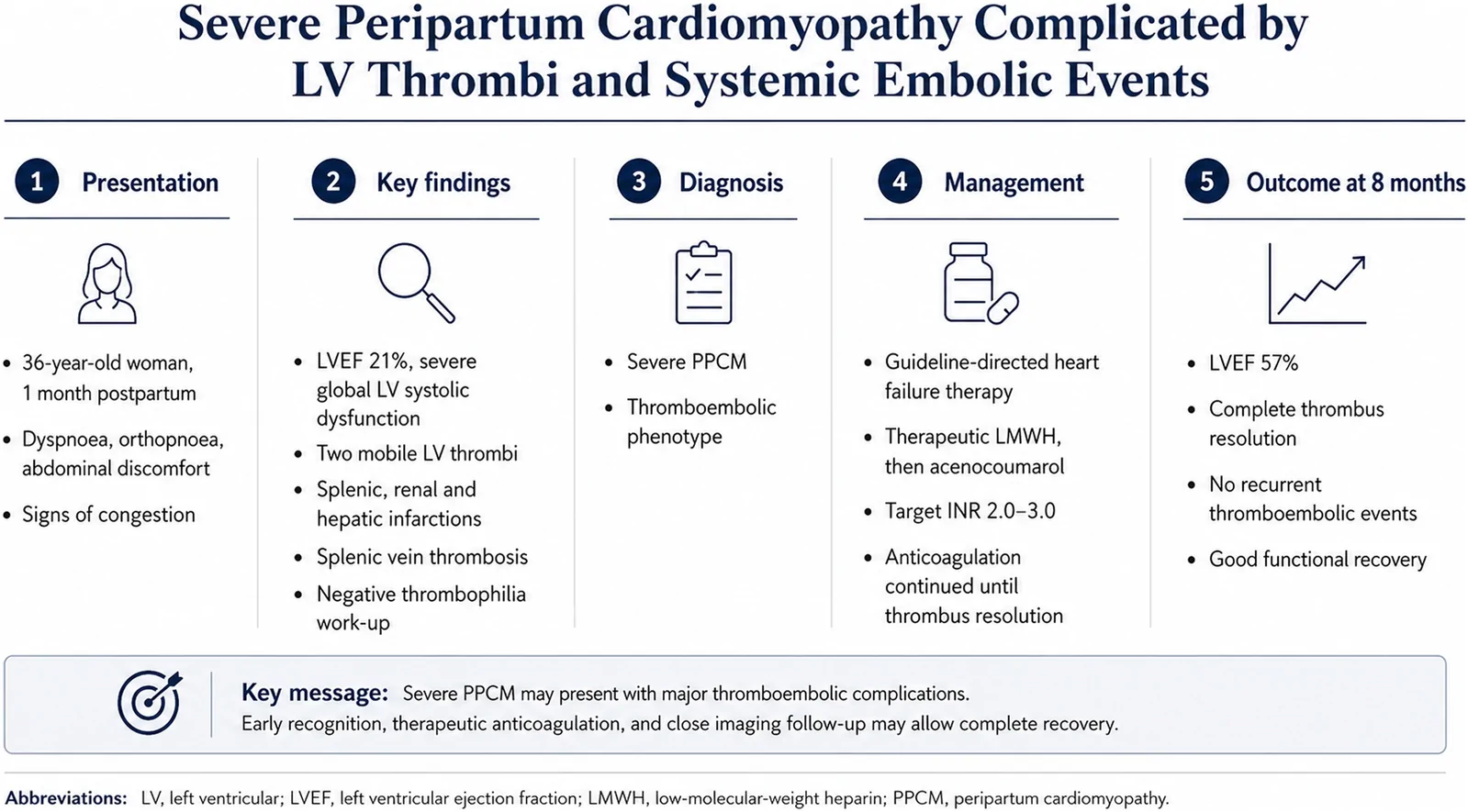


## Case presentation

A 36-year-old woman (G3P3) was admitted 1 month postpartum for acute decompensated heart failure. She had no cardiovascular risk factors, no known structural heart disease, and no family history suggestive of inherited cardiomyopathy. During her most recent pregnancy, she had developed progressively worsening exertional dyspnoea from the fifth month of gestation, associated with gradual functional limitation. These symptoms were initially attributed to physiological changes of pregnancy and were not investigated. There was no history of pre-eclampsia, hypertensive disorder of pregnancy, or peripartum infectious complication. Delivery was uncomplicated, and the immediate postpartum course was initially uneventful.

One month after delivery, she presented with worsening dyspnoea, orthopnoea, and diffuse abdominal discomfort. On admission, she was tachycardic at 130 b.p.m. and hypertensive, with oxygen saturation of 88% on room air, improving with low-flow oxygen therapy. Physical examination showed signs of global congestion, including bilateral lower-limb oedema, jugular venous distension, reduced breath sounds at both lung bases consistent with pleural effusions, and ascites. There were no clinical signs of cardiogenic shock or peripheral hypoperfusion.

**Figure 1:**
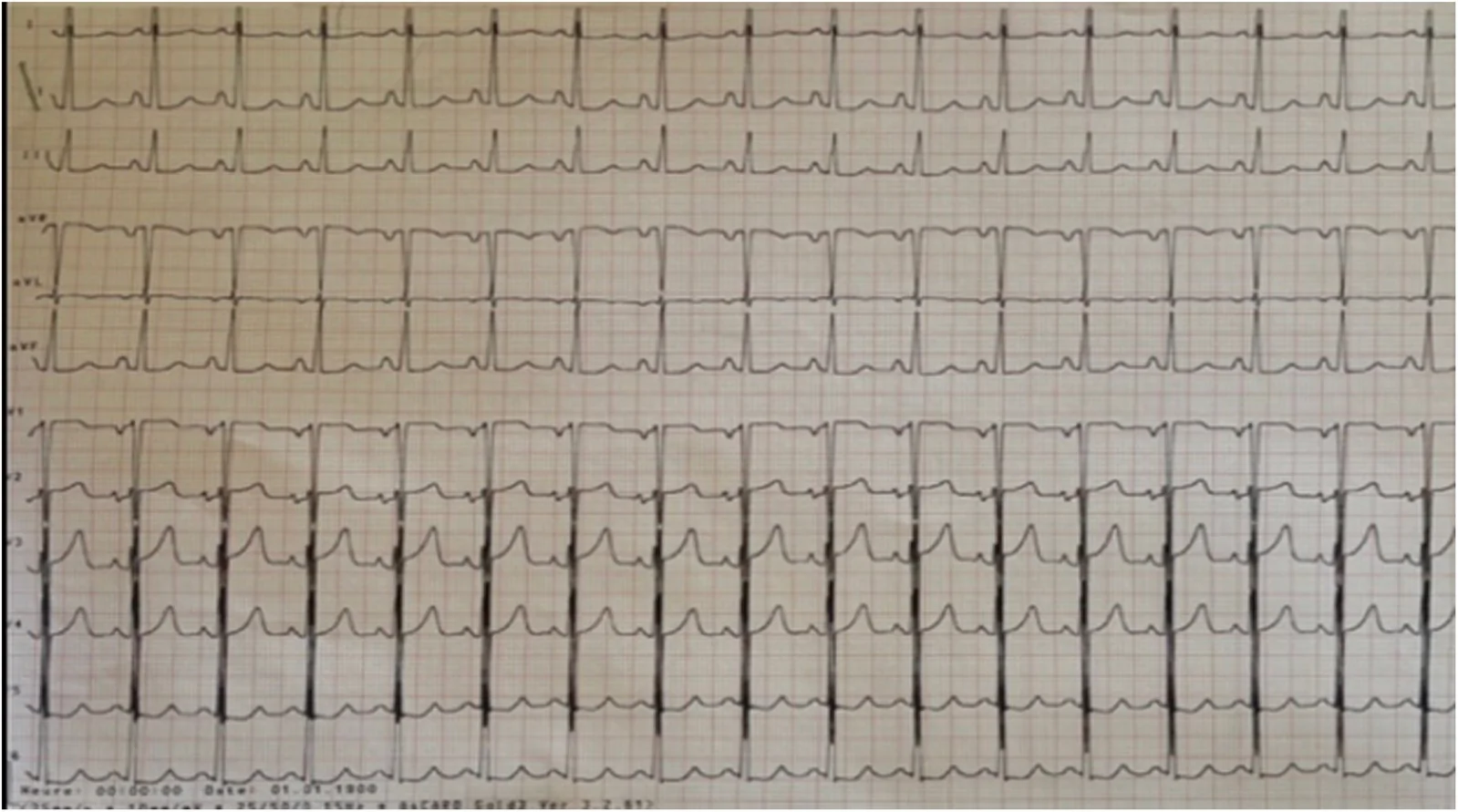
Admission electrocardiogram. Admission 12-lead electrocardiogram demonstrating sinus tachycardia with voltage criteria for left ventricular hypertrophy and no acute ischaemic ST-segment abnormalities.

Electrocardiography showed sinus tachycardia with voltage criteria for LV hypertrophy, without acute ischaemic ST-segment changes (*Figure 1*). Chest radiography demonstrated marked cardiomegaly, bilateral pleural effusions, and pulmonary oedema.

**Figure 2:**
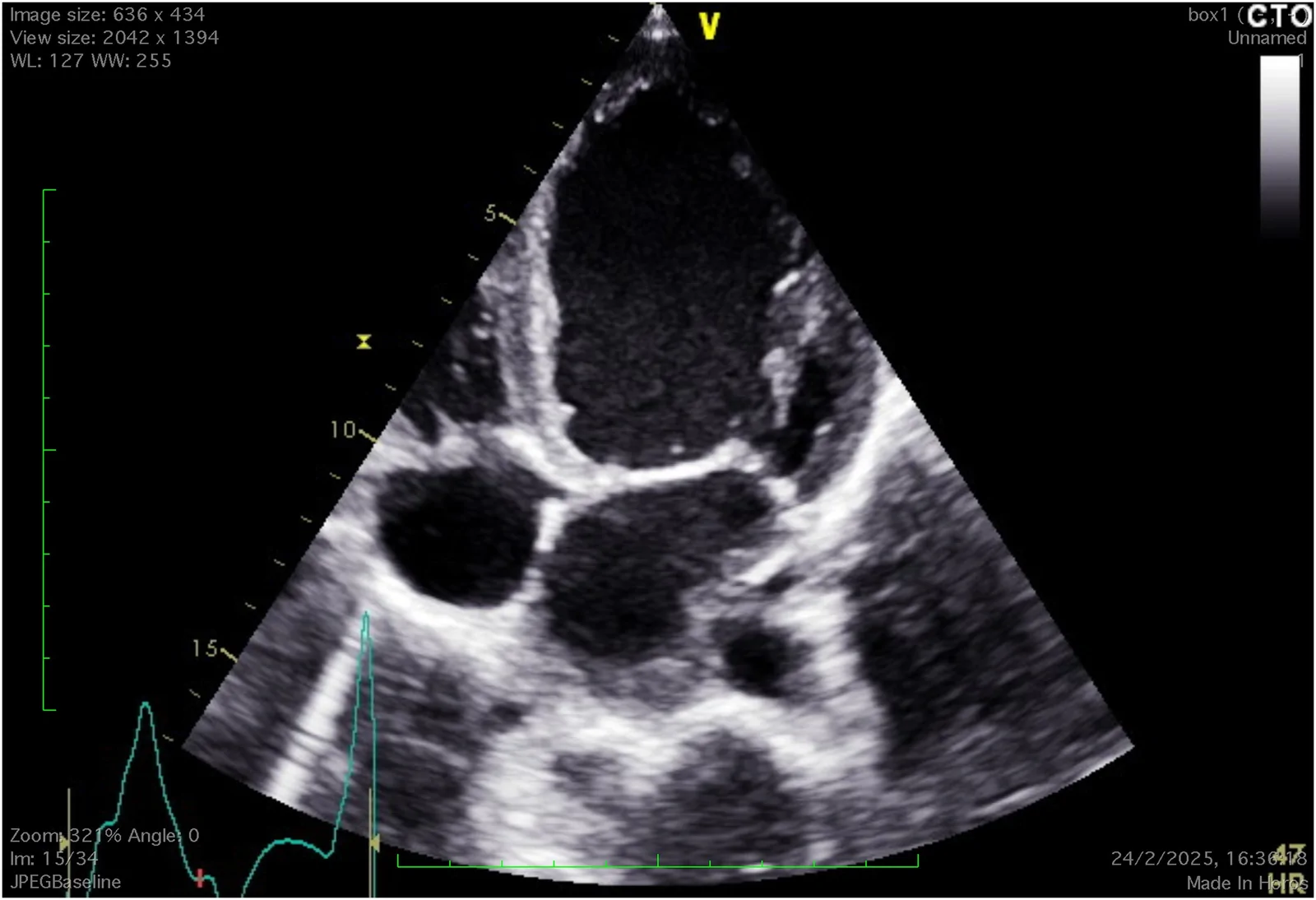
Initial transthoracic echocardiography. Apical four-chamber transthoracic echocardiographic view obtained at presentation showing marked left ventricular dilatation in the context of severe global systolic dysfunction. Left ventricular ejection fraction was estimated at 21% by the Simpson biplane method. No regional wall-motion abnormality was identified, and right ventricular systolic function was preserved.

Transthoracic echocardiography revealed a markedly dilated left ventricle with severe global hypokinesia and a LV ejection fraction (LVEF) of 21%, assessed by the Simpson biplane method. A restrictive transmitral filling pattern suggested elevated LV filling pressures. No regional wall-motion abnormality was identified. Right ventricular size and systolic function were preserved, without significant pulmonary hypertension. Mild functional mitral regurgitation was present, and the pericardium appeared normal (*Figure 2*; Supplementary material online, *Video S1*). Continuous in-hospital rhythm monitoring, performed because of the severity of LV dysfunction, did not reveal sustained ventricular arrhythmias.

**Figure 3:**
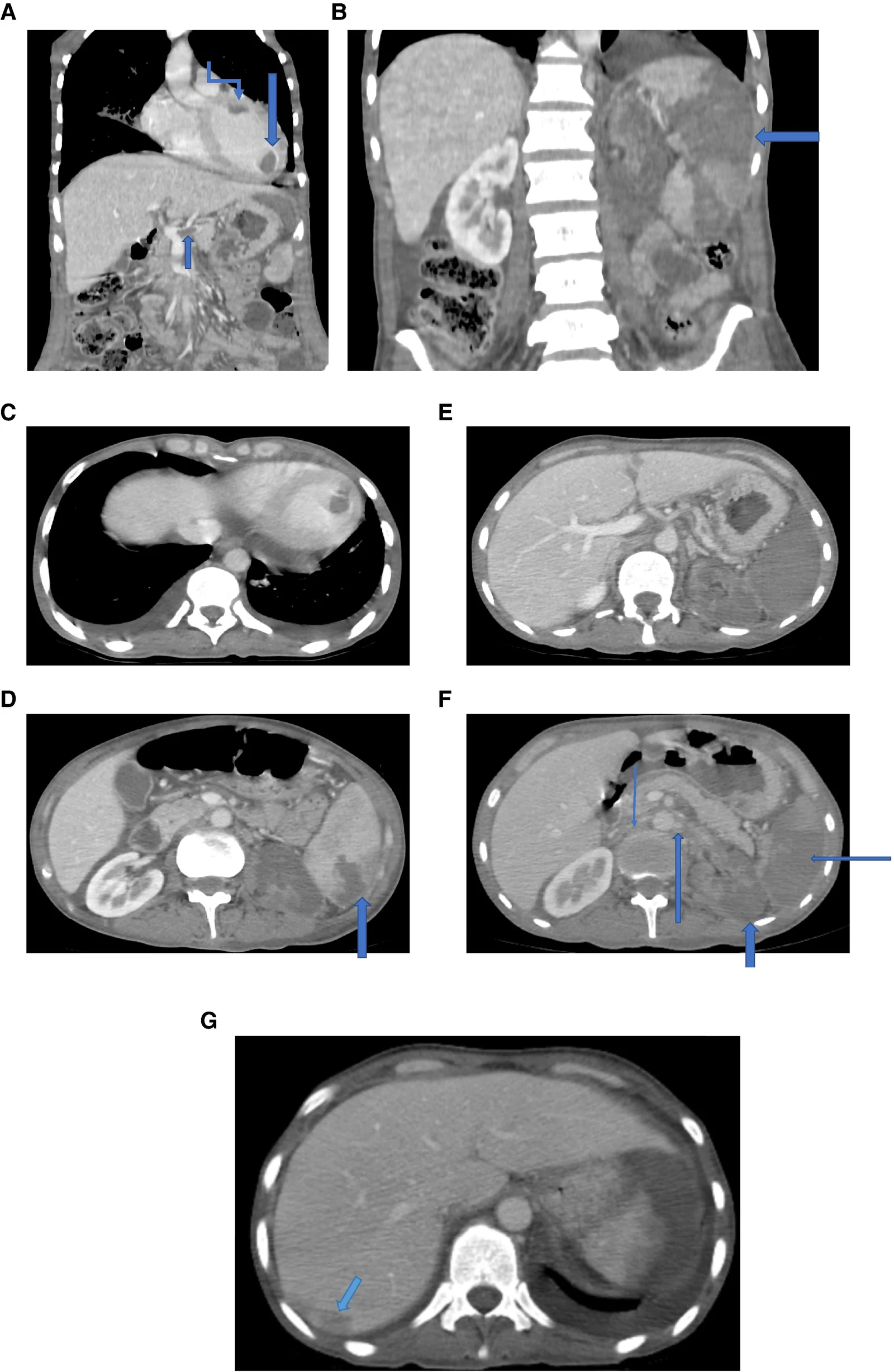
Computed tomography findings at diagnosis. Multiplanar contrast-enhanced thoracoabdominopelvic computed tomography demonstrating intracardiac thrombi and widespread systemic thromboembolic complications. (*A*) Coronal thoracoabdominal computed tomography image demonstrating two intracavitary left ventricular thrombi and splenic vein thrombosis extending from its origin. (*B*) Coronal abdominal computed tomography image demonstrating an enlarged left kidney with multiple peripheral wedge-shaped hypodense lesions, consistent with renal infarctions, with residual enhancing parenchyma after contrast administration. (*C*) Axial thoracic computed tomography image demonstrating a hypodense intracavitary mass within the left ventricular apex, consistent with thrombus. (*D*) Axial abdominal computed tomography image demonstrating near-complete hypodensity of the left renal parenchyma with peripheral capsular enhancement, consistent with extensive renal infarction, with associated splenic infarctions. (*E*) Axial abdominal computed tomography image demonstrating partial splenic vein thrombosis associated with extensive renal and splenic infarctions. (*F*) Axial contrast-enhanced computed tomography image showing bilateral renal and splenic infarctions. The right renal artery appears markedly attenuated, whereas only the ostium of the left renal artery is opacified, with absent opacification of the remaining left renal vascular pedicle. (*G*) Axial contrast-enhanced abdominal computed tomography image demonstrating a peripheral subcapsular hypodense area involving hepatic segments VI and VII, without post-contrast enhancement, consistent with hepatic infarction. Blue arrows indicate the key pathological findings described in panels A, B, D, F, and G.

In the context of hypoxaemia and elevated D-dimer levels, computed tomography (CT) pulmonary angiography was performed to exclude pulmonary embolism. Pulmonary embolism was not identified. The scan confirmed pulmonary oedema and demonstrated intracardiac thrombi, including two large LV thrombi: A peri-septal thrombus measuring 22 × 10 mm and an apical thrombus measuring 16 × 12 mm. Additional thoracoabdominal imaging revealed multiple systemic embolic events, including splenic, renal, and hepatic infarctions, as well as splenic vein thrombosis. Moderate-to-large ascites and bilateral pleural effusions were also noted (*Figure 3*).

Laboratory testing showed a markedly elevated B-type natriuretic peptide level (1430 pg/ml; reference <100 pg/ml). High-sensitivity cardiac troponins were repeatedly negative. White blood cell count and procalcitonin levels were normal, and no infectious focus was identified. C-reactive protein was elevated at 107 mg/l in the absence of fever or microbiological evidence of infection. Mild iron-deficiency anaemia was present (haemoglobin 11.5 g/dl). Liver enzymes were transiently elevated, consistent with congestive hepatopathy and/or hepatic infarction, and improved following decongestion. Renal and thyroid function were normal.

Alternative causes of cardiomyopathy were systematically considered. Acute coronary syndrome was considered unlikely given the absence of chest pain, repeatedly negative high-sensitivity cardiac troponins, and the absence of regional wall-motion abnormalities. Stress-induced cardiomyopathy was also considered unlikely because of the absence of a typical regional contractile pattern. There was no history of pre-existing structural heart disease, chronic hypertension, pre-eclampsia, hypertensive disorder of pregnancy, substance exposure, or peripartum infectious complication. The absence of fever, normal procalcitonin level, absence of an infectious focus, repeatedly negative cardiac biomarkers, and global rather than regional LV dysfunction made active myocarditis less likely, although cardiac magnetic resonance imaging was not available for tissue characterization. A comprehensive thrombophilia work-up performed outside the acute phase showed normal protein C, protein S, and antithrombin levels and was negative for factor V Leiden mutation, prothrombin gene mutation, and antiphospholipid antibodies.

Cardiac magnetic resonance imaging was not performed because of financial constraints, which represents a diagnostic limitation. However, the temporal relationship with pregnancy, absence of previous structural heart disease, severe global rather than regional LV systolic dysfunction, repeatedly negative cardiac biomarkers, lack of evidence for an alternative cause, and subsequent complete recovery during follow-up were considered most consistent with PPCM.

Intravenous loop diuretics were initiated, resulting in rapid clinical improvement and progressive decongestion. After haemodynamic stabilization, guideline-directed medical therapy for heart failure with reduced ejection fraction was introduced, including an angiotensin-converting enzyme inhibitor, beta-blocker, and mineralocorticoid receptor antagonist. Therapeutic anticoagulation was started with weight-adjusted low-molecular-weight heparin during the acute phase and subsequently transitioned to acenocoumarol, with a target international normalized ratio of 2.0–3.0. Anticoagulation was continued until complete resolution of LV thrombi on follow-up imaging. Breastfeeding was discontinued, and progestin-only contraception was initiated before discharge.

**Figure 4:**
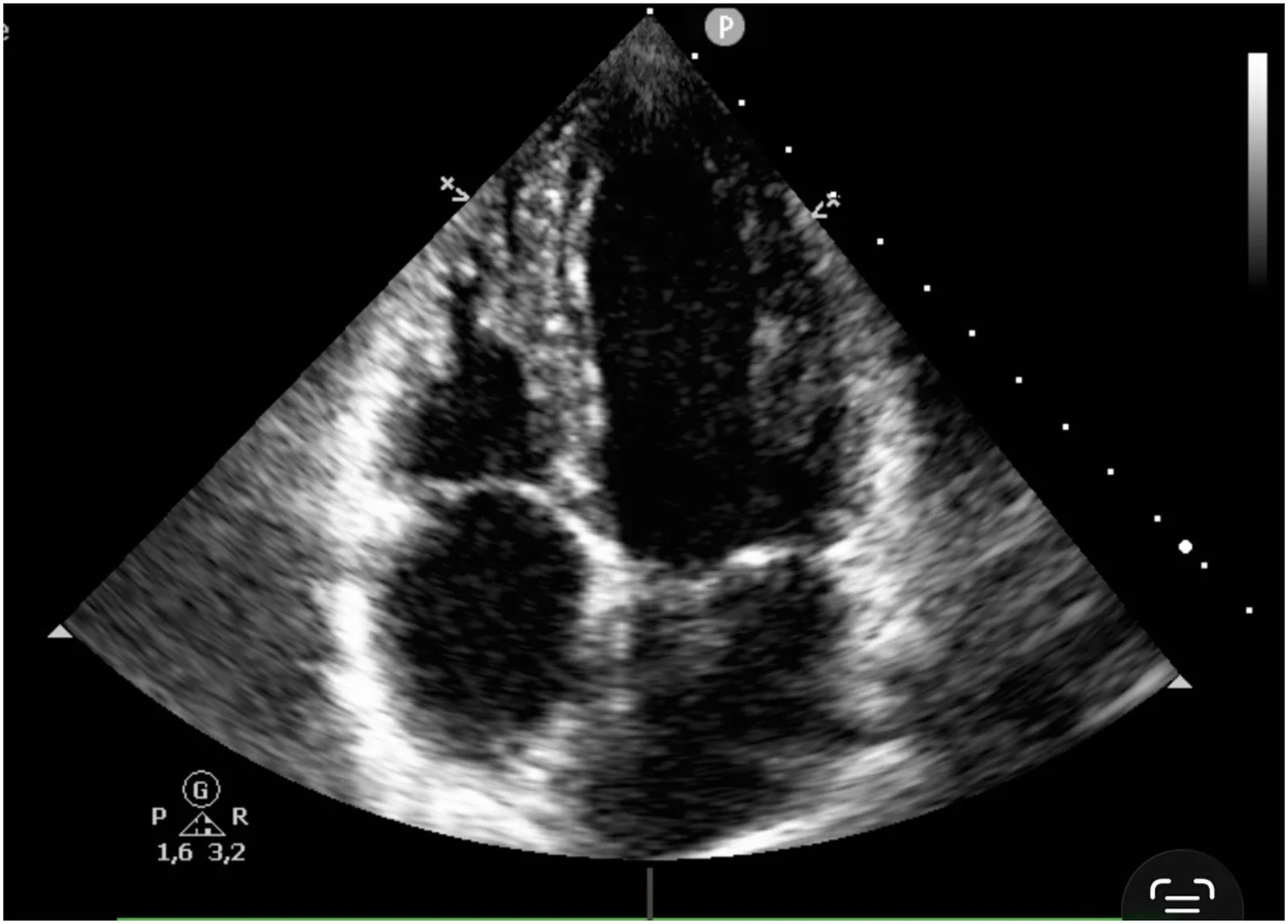
Follow-up transthoracic echocardiography after recovery. Follow-up transthoracic echocardiography at 8 months, obtained in the apical four-chamber view, demonstrating complete resolution of the previously documented left ventricular thrombi, normalization of left ventricular cavity appearance, and marked recovery of left ventricular systolic function, with left ventricular ejection fraction improving to 57%.

The patient was discharged after 17 days of hospitalization. At 1 month, she had substantial clinical improvement, with a reduction of symptoms to New York Heart Association functional Class II. Echocardiography showed early reverse remodelling, with LVEF improving to 35%. The previously documented LV thrombi had markedly decreased in size but remained visible as residual mural thrombotic images. At 3 months, continued improvement in LV systolic function was associated with further thrombus regression and no recurrent thromboembolic event. At 6 months, LVEF had improved to 50%, and only minimal residual thrombotic images persisted. Anticoagulation was therefore maintained until complete thrombus resolution was confirmed. At 8 months, LVEF had normalized to 57%, with complete disappearance of LV thrombi and no recurrent systemic embolic event (*Figure 4*; Supplementary material online, *Video S2*).

## Discussion

The present observation raises a broader issue in the management of PPCM: the extent to which severe ventricular dysfunction in the early postpartum period should be regarded not only as a haemodynamic disorder but also as a condition with substantial thromboembolic potential. This distinction is clinically important because thromboembolic complications may be silent, atypical, or extracardiac at presentation, and their recognition can substantially influence diagnostic strategy, anticoagulation decisions, and follow-up intensity. In this context, the case illustrates a severe thromboembolic phenotype of PPCM, in which intracardiac thrombosis, multivisceral embolization, and venous thrombosis occurred despite the absence of an identified inherited or acquired thrombophilia.^3,8^

The frequency of thromboembolic events in PPCM is variable across studies, reflecting differences in diagnostic criteria, timing of presentation, severity of LV dysfunction, imaging practices, and anticoagulation use.^3,8^ In the ESC EURObservational Research Programme (EORP) PPCM Registry, thromboembolic events were reported in ∼5% of women during the index hospitalization.^3^ In the 1-year EORP outcomes analysis, thromboembolism and stroke occurred in 6.3% and 2.5% of patients, respectively, while LV recovery had not occurred in approximately one-third of women at 1 year.^9^ These data indicate that thromboembolic events affect a minority of patients with PPCM but remain clinically important because they contribute to morbidity and often occur in the setting of severe LV dysfunction, a recognized marker of adverse outcome.^3,9^

The mechanistic basis for thrombosis in PPCM is multifactorial and particularly relevant to the present patient. Severe LV systolic dysfunction and LV dilatation promote intracavitary stasis, favouring thrombus formation in low-flow regions of the LV cavity. This local haemodynamic substrate occurs during a period already characterized by increased thrombotic tendency related to pregnancy and the puerperium, with activation of coagulation pathways and reduced fibrinolytic activity. Endothelial dysfunction, inflammatory activation, systemic congestion, and reduced mobility during acute heart failure may further amplify this risk.^3,8^ The EORP thromboembolism analysis emphasized LV dilatation, endothelial injury, immobility, and postpartum hypercoagulability as contributors to thromboembolism in PPCM.^3^ The systematic review by Radakrishnan *et al*. also highlighted low LVEF, altered coagulation factors, reduced fibrinolysis, and heterogeneity in anticoagulation strategies as key elements in PPCM-related thromboembolism.^8^ In our patient, these mechanisms converged in a particularly unfavourable way: severe LV systolic impairment created the intracardiac substrate for thrombus formation, while the postpartum prothrombotic state likely facilitated both embolic dissemination and venous thrombosis.

This dual arterial and venous thrombotic expression is one of the most instructive aspects of the case. Renal, splenic, and hepatic infarctions were compatible with systemic embolization from LV thrombi, whereas splenic vein thrombosis suggested a broader prothrombotic milieu rather than a single isolated embolic event. The negative thrombophilia work-up performed outside the acute-phase strengthens the interpretation that the thrombotic burden was driven primarily by the interaction between severe PPCM-related haemodynamic impairment and postpartum haemostatic vulnerability. This point is clinically relevant because extensive thrombosis in PPCM should not automatically be attributed to an underlying thrombophilia; severe PPCM itself may be sufficient to generate a high-risk thrombotic environment.^3,8^

The published literature supports the possibility of visceral embolization in PPCM, although the number of comparable reports remains limited. Ibebuogu *et al*. described PPCM presenting with multiple thromboembolic phenomena involving abdominal organs.^4^ Manikkan and Sanati reported splenic infarction as the presenting manifestation of PPCM.^5^ Wahab and Tariq described splenic and renal infarction associated with venous thromboembolism in PPCM.^6^ More recently, Yao *et al*. reported renal and splenic infarctions complicating PPCM in association with LV thrombus.^7^ Compared with these observations, the present case appears to occupy the severe end of the reported spectrum, combining two mobile LV thrombi, bilateral renal infarctions, splenic and hepatic infarctions, and splenic vein thrombosis. The case is therefore instructive not only because of its rarity but also because it brings together several mechanisms and complications that are usually described separately.

The diagnostic challenge lies in the fact that visceral embolization may mimic, or be masked by, manifestations of acute heart failure. Abdominal discomfort, ascites, hepatic enzyme elevation, pleural effusions, and systemic congestion may all be explained by decompensated heart failure, yet they may coexist with embolic injury in the same patient. Published cases of PPCM presenting with acute abdomen, splenic infarction, or renal and splenic infarctions show that extracardiac embolic events may be clinically misleading and may remain unrecognized without targeted imaging.^4–7^ In this setting, CT has a complementary role to echocardiography: echocardiography characterizes ventricular function and intracardiac thrombus, while CT can identify extracardiac embolic complications and exclude pulmonary embolism when clinically suspected. The present case therefore supports a low threshold for multimodality imaging when abdominal symptoms are persistent, atypical, or disproportionate to the apparent degree of congestion in severe PPCM.^10^

The diagnosis of PPCM in this patient was supported by a convergent diagnostic argument rather than by a single confirmatory test. Peripartum cardiomyopathy remains a diagnosis of exclusion, requiring the absence of pre-existing structural heart disease or another identifiable cause of heart failure. In the present case, the temporal relationship with pregnancy, the severe global rather than regional pattern of LV systolic dysfunction, the absence of acute ischaemic evidence, and the subsequent complete recovery were all consistent with PPCM.^1,2^ The differential diagnosis included acute coronary syndrome, stress-induced cardiomyopathy, myocarditis, pre-existing dilated cardiomyopathy, hypertensive heart disease, and other non-ischaemic cardiomyopathies.^1,2,10^ Rather than excluding each entity through a single test, the overall diagnostic probability was shaped by the clinical context, biomarker profile, echocardiographic pattern, absence of an alternative cause, and recovery trajectory. Cardiac magnetic resonance imaging would have strengthened this assessment by providing tissue characterization and improving the evaluation of myocarditis, Takotsubo syndrome, intracardiac thrombus, and other non-ischaemic cardiomyopathies, and its absence remains a limitation.^10^

Therapeutic management in severe PPCM should address two parallel objectives: ventricular recovery and thromboembolic protection. Guideline-directed heart failure therapy was introduced after haemodynamic stabilization, as improvement in LV function and reduction of intracavitary stasis are themselves central to reducing thromboembolic risk.^11^ Antithrombotic management in PPCM, however, remains one of the least standardized aspects of care, largely because high-quality prospective data are lacking and recommendations rely mainly on expert consensus, observational evidence, and extrapolation from other forms of LV thrombus. In patients without documented thrombosis, the decision to anticoagulate is generally risk-based, with proposed thresholds centred on severe LV systolic dysfunction, most commonly LVEF <30% or ≤35%, particularly during the early postpartum period or when bromocriptine is used. This uncertainty reflects the difficulty of balancing embolic risk against bleeding risk in a population whose thrombotic risk is dynamic and influenced by both cardiac and postpartum haemostatic factors.^8,11^

This uncertainty is substantially reduced when intracardiac thrombus or systemic embolism is present. In that setting, the therapeutic objective is no longer primary prevention but secondary prevention of recurrent embolization, and full-dose anticoagulation is generally supported.^8,11^ The present patient belonged to this high-risk category because LV thrombi were already documented and multivisceral embolic events had occurred. The choice of initial weight-adjusted low-molecular-weight heparin was therefore consistent with the need for immediate therapeutic anticoagulation in the acute phase, when rapid onset, predictable anticoagulant effect, and clinical flexibility are important.^11^ After haemodynamic stabilization, transition to acenocoumarol with a target international normalized ratio of 2.0–3.0 allowed sustained outpatient anticoagulation during the period of ongoing LV recovery and thrombus regression.

The choice of long-term anticoagulant in postpartum PPCM should also be interpreted in the light of the available evidence. Heparin-based regimens, including unfractionated heparin and low-molecular-weight heparin, have the most established role around pregnancy and the early postpartum period, whereas vitamin K antagonists are commonly used after delivery when prolonged anticoagulation is required.^11^ Direct oral anticoagulants have been used postpartum in some contexts, but evidence supporting their safety and efficacy specifically for PPCM-associated LV thrombus remains limited.^8^ In this case, the use of acenocoumarol rather than a direct oral anticoagulant was therefore a conservative and evidence-aligned strategy, particularly given the presence of large mobile thrombi and systemic embolization.

Duration of anticoagulation is another unresolved issue. No randomized data define a fixed treatment duration for PPCM complicated by LV thrombus, and practice varies across centres and healthcare systems. For this reason, discontinuation should not be based solely on symptomatic improvement or partial recovery of LVEF. A more rational approach is to integrate serial imaging, thrombus resolution, degree of LV recovery, embolic recurrence, and bleeding risk.^8,11^ In the present case, residual mural thrombotic images persisted despite early clinical improvement and progressive LV reverse remodelling. Anticoagulation was therefore maintained until complete disappearance of LV thrombi, which occurred in parallel with normalization of LVEF at 8 months. This imaging-guided strategy illustrates a pragmatic approach to secondary thromboembolic prevention in severe PPCM, in which treatment duration is individualized according to the evolution of both the thrombotic substrate and ventricular function.

Although bromocriptine was not used in the present patient, it remains an important but nuanced therapeutic consideration in PPCM. Its biological rationale is based on prolactin inhibition and prevention of the generation of a 16 kDa prolactin fragment implicated in endothelial dysfunction, impaired angiogenesis, inflammation, and myocardial injury.^12^ In the multicentre randomized BB-EFFECT study, both short- and longer-term bromocriptine regimens were associated with high rates of LV recovery in severe PPCM, although the study did not include a placebo arm and was not powered to demonstrate a mortality benefit.^13^ More recent EORP data suggested that bromocriptine treatment was associated with better maternal outcome at 6 months, mainly driven by fewer patients with persistent severe LV dysfunction; however, these findings came from an exploratory, non-randomized analysis and remain subject to residual confounding and treatment-selection bias.^14^ Bromocriptine should therefore be discussed as a potential adjunctive therapy in selected patients with severe PPCM rather than as a universally required treatment.^13,14^ When used, it should be accompanied by anticoagulation because of both the thrombotic context of PPCM and the need to mitigate potential treatment-related thrombotic risk.^11,13^

The favourable outcome in this case should be interpreted in the light of the heterogeneous prognosis of PPCM. Severe LV dysfunction at diagnosis is generally considered an adverse feature, and EORP 1-year data show that a substantial proportion of patients does not achieve LV recovery at 1 year.^9^ However, prognosis is also influenced by early response to therapy, LV reverse remodelling, and right ventricular involvement.^2,9^ In this patient, preserved right ventricular systolic function and early improvement in LVEF may have contributed to the favourable trajectory. The progressive increase in LVEF from 21% at presentation to 57% at 8 months, together with complete thrombus resolution and absence of recurrent embolic events, illustrates that even an extensive thromboembolic presentation does not necessarily imply irreversible myocardial injury. This distinction is important for counselling and follow-up, as the initial embolic burden reflects acute disease severity but does not by itself determine the reversibility of myocardial dysfunction.

Long-term management after apparent recovery should also include reproductive counselling and contraception planning. Subsequent pregnancy after PPCM carries a risk of recurrent LV dysfunction, particularly when LV recovery is incomplete, and is not recommended if LV function does not normalize. Even in women with recovered LV function, relapse remains possible, making specialist preconception counselling and close cardio-obstetric follow-up essential before any future pregnancy. In the present patient, progestin-only contraception was initiated before discharge. This choice was consistent with the recent severe thromboembolic presentation, as oestrogen-containing contraception may increase thrombotic risk, whereas progestin-only strategies have a more favourable cardiovascular and haemostatic profile.^11^

## Conclusion

Severe PPCM should be considered not only as a heart failure syndrome but also as a condition with substantial thromboembolic potential. In patients with markedly reduced LV ejection fraction, intracardiac thrombus, or atypical extracardiac symptoms, systematic assessment for both cardiac and extracardiac thromboembolic complications is warranted. When LV thrombus or systemic embolization is present, anticoagulation should be therapeutic and guided by serial imaging, thrombus resolution, ventricular recovery, and bleeding risk. Early recognition, guideline-directed heart failure therapy, individualized anticoagulation, and close imaging follow-up may allow complete recovery even after an initially severe thromboembolic presentation.

## Lead author biography


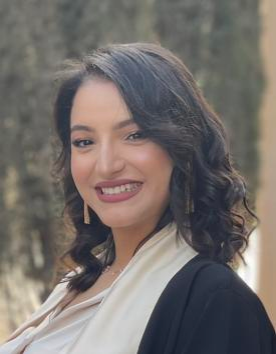
Dr Fadoua Achour, a cardiology resident at the University Hospital Centre of Rabat, Morocco. Her daily hospital practice has gradually led her to develop an interest in clinical research, especially through the recognition of unusual and educational cases. She is particularly interested in heart failure and the critical analysis of complex clinical presentations. Through research and case-based learning, she hopes to contribute to a more thoughtful and evidence-based approach to cardiovascular care.

## Supplementary Material

ytag685_Supplementary_Data

## Data Availability

All relevant data are included within the article.
